# The Recovery of Vanadium Pentoxide (V_2_O_5_) from Spent Catalyst Utilized in a Sulfuric Acid Production Plant in Jordan

**DOI:** 10.3390/ma16196503

**Published:** 2023-09-30

**Authors:** Hiba H. Al Amayreh, Aya Khalaf, Majd I. Hawwari, Mohammed K. Hourani, Abeer Al Bawab

**Affiliations:** 1 Department of Scientific Basic Sciences, Faculty of Engineering Technology, Al-Balqa Applied University, Amman 11134, Jordan; hiba.alamyreh@bau.edu.jo; 2 Department of Basic Sciences, Faculty of Arts and Sciences, Al-Ahliyya Amman University, Amman 19328, Jordan; a.khaled@ammanu.edu.jo; 3 Jordan Atomic Energy Commission, Amman 11934, Jordan; majd.hawari@gmail.com; 4 Chemistry Department, School of Science, The University of Jordan, Amman 11942, Jordan; mhourani@ju.edu.jo; 5 Hamdi Mango Center for Scientific Research, The University of Jordan, Amman 11942, Jordan

**Keywords:** vanadium pentoxide, leaching process, recovery of V_2_O_5_, recycling, extraction

## Abstract

Vanadium is a significant metal, and its derivatives are widely employed in industry. One of the essential vanadium compounds is vanadium pentoxide (V_2_O_5_), which is mostly recovered from titanomagnetite, uranium–vanadium deposits, phosphate rocks, and spent catalysts. A smart method for the characterization and recovery of vanadium pentoxide (V_2_O_5_) was investigated and implemented as a small-scale benchtop model. Several nondestructive analytical techniques, such as particle size analysis, X-ray fluorescence (XRF), inductively coupled plasma (ICP), and X-ray diffraction (XRD) were used to determine the physical and chemical properties, such as the particle size and composition, of the samples before and after the recovery process of vanadium pentoxide (V_2_O_5_). After sample preparation, several acid and alkali leaching techniques were investigated. A noncorrosive, environmentally friendly extraction method based on the use of less harmful acids was applied in batch and column experiments for the extraction of V_2_O_5_ as vanadium ions from a spent vanadium catalyst. In batching experiments, different acids and bases were examined as leaching solution agents; oxalic acid showed the best percent recovery for vanadium ions compared with the other acids used. The effects of the contact time, acid concentration, solid-to-liquid ratio, stirring rate, and temperature were studied to optimize the leaching conditions. Oxalic acid with a 6% (*w*/*w*) to a 1/10 solid-to-liquid ratio at 300 rpm and 50 °C was the optimal condition for extraction (67.43% recovery). On the other hand, the column experiment with a 150 cm long and 5 cm i.d. and 144 h contact time using the same leaching reagent, 6% oxalic acid, showed a 94.42% recovery. The results of the present work indicate the possibility of the recovery of vanadium pentoxide from the spent vanadium catalyst used in the sulfuric acid industry in Jordan.

## 1. Introduction

Vanadium, the 22nd most prevalent element, is a metal of great value that is found extensively in the Earth’s crust [1]. Vanadium is mostly used in ferrovanadium or as a steel-enhancing agent and accounts for 85% of all vanadium output [2]. Among the numerous vanadium compounds, the oxide of pentavalent vanadium (V_2_O_5_) is frequently utilized as a catalyst in diverse chemical transformations and represents an important industrial usage of vanadium in addition to that in steel [3].

The recovery of vanadium pentoxide (V_2_O_5_) from a spent catalyst is of prime importance from industrial, economic, and environmental standpoints [4]. Vanadium pentoxide (V_2_O_5_) represents a material of prime importance that has important technological applications due to its spectacular electronic, magnetic, and catalytic properties [5]. The major use of vanadium pentoxide (V_2_O_5_) is as a catalyst in the manufacturing of sulphuric acid, which can be used in oil refining, treatment of steel, production of phosphoric acid, the later can be used in production of detergents, soap, and fertilizers, in addition to many other applications [6]. Vanadium pentoxide (V_2_O_5_) supported on a silica substrate is used as a catalyst in the contact method in the manufacturing of sulfuric acid where SO_2_ is converted into SO_3_ [7]. Huge quantities of spent catalysts, however, are accumulated in the environment as industrial waste [8]. Accumulation of the spent vanadium catalyst, however, poses a real environmental problem [9]. The air above the accumulated waste is loaded with vanadium pentoxide dust and transferred to the neighboring areas by wind. Vanadium pentoxide is known to have adverse effects on humans, animals, and plants [10]. Vanadium pentoxide causes nausea, vomiting, salivation, lacrimation, and the absence of a pulse in people and animals. Inhaling vanadium pentoxide (V_2_O_5_) causes DNA base oxidation, DNA repair, and the production of micronuclei, nucleoplasmic bridges, and nuclear buds. The workers may be more susceptible to cancer and other disorders linked to DNA instability. In human lymphocytes, it causes single-strand DNA breakage. Vanadium pentoxide (V_2_O_5_) exposure can weaken lung resistance to infections. Some of the health threats of V_2_O_5_ for humans include nausea, vomiting, the disappearance of pulse, and the risk of lung cancer [11]. These health issues make the recovery of V_2_O_5_ from spent catalyst waste an important subject for this research project.

The recovery of vanadium pentoxide (V_2_O_5_) from spent vanadium catalysts via chemical methods has been reported by many researchers [2,3,4,5,6]. In past investigations, different leaching strategies had been applied to liberate V_2_O_5_ from the support matrix [8,9]. Sulfuric acid, citric acid, oxalic acid, (NH_4_) S_2_O_3_, hydrogen peroxide was used as leaching agents under different experimental conditions [4,12,13].

Several studies have investigated the effectiveness of the recovery of V_2_O_5_ from spent vanadium catalysts. Li et al. designed the direct acid leaching approach, which is used to extract vanadium from a variety of solid sources. Under pressure from oxygen, the vanadium recovery yield increased to 80% [14]. A similar technique was used by Deng et al. to recover vanadium from unburned stone coal, and they obtained a recovery yield of more than 90% [15]. Shi et al. reported a vanadium recovery of approximately 67.7% in their investigations on the recovery of vanadium from black shale utilizing ultrasonic leaching [16]. On the other hand, it is worth mentioning that some studies provide flotation procedures as a useful technique for the recovery of metals from ores and other sources of metal [17,18].

The quest for new or improved leaching methods for the recovery of V_2_O_5_ is still a hot topic in chemical research because of its relationship with public health, the environment, and the economy [19,20,21]. Many studies have been performed on other wastes focusing on environmental issues, such as olive mill wastewater (OMW) [22,23] and textile wastewater [24,25,26]. In Jordan, to our knowledge, this is the first study performed on spent catalysts and on spent catalyst leaching using a solid spent catalyst as the batch and column scale together with a weak acid or base. The present work is necessary to provide a startup project that utilizes the remediation process of vanadium recovery from a spent catalyst. This work was undertaken with the goal of leaching the maximum amount of V_2_O_5_ from the spent vanadium catalyst and optimizing the experimental conditions to pursue this task. Moreover, this work aimed to choose an environmentally friendly or nearly environmentally friendly leaching reagent with the fewest adverse effects on the environment.

## 2. Materials and Methods

### 2.1. Materials

The following chemicals were used as-received from suppliers without further purification. Oxalic acid (C_2_H_2_O_4_) (extra pure) was purchased from Scharlau (Barcelona, Spain); sulfuric acid (H_2_SO_4_) (99.9% purity) and acetic acid (CH_3_COOH) (99.9% purity) were supplied by Merck (Rahway, NJ, USA); potassium hydroxide (KOH) (98% purity) was from Riedel-de Haën (Seelze, Germany); citric acid (C_6_H_8_O_7_) (Extra pure) and hydrogen peroxide (H_2_O_2_) (37 wt.%) were supplied by Labo Chemie (Mumbai, India); vanadium pentoxide (V_2_O_5_) was supplied by BDH Chemical Ltd. (Poole, UK).

### 2.2. Preparation and Characterization of Spent Vanadium Catalyst Samples

The spent vanadium catalyst was supplied by the Jordan Phosphate Mines Company (JPMC, Maan, Jordan). The catalyst was provided by the company after the turnover finished, and it was withdrawn as waste. The spent vanadium catalyst samples were composed of 6 mm average diameter with 20 mm length hexagonal prism structures. The samples were ground by using a planetary ball mill and sieved to isolate particles of ≤50 µm diameter in the laboratories of Jordan Atomic Energy Commission. Ground samples (mass = 5–25 g) were used in batch experiments, keeping the used volume of leaching acid at 50.0 mL, while 2.40 kg of underground solid samples was used in column experiments with the following dimensions: 150 cm long and 5 cm internal diameter.

Spent catalyst samples were characterized via Zeta potential analysis using Zetasizer, Nano-ZS (Malvern Instruments Ltd., Malvern, UK). Particle size/charge analysis and zeta potential Zetatrac analyzer (Microtrac, York, PA, USA) were used for zeta potential. Particle size analysis and X-ray diffraction XRD (MAXIMA 7000 and X-ray spectrophotometer (Shi-madzu, Tokyo, Japan) were used for the XRD pattern. XRF data were analyzed via X-ray fluorescence XRF, lab center XRF-1800 sequential, and X-ray fluorescence spectrophotometer (Shimadzu, Tokyo, Japan). ICP analysis (ICP-AES GBC E1475, New York, NY, USA) was used for V-ion concentration analysis.

The XRD pattern of the fresh catalyst before industrial use and spent catalysts after the catalytic cycle and leaching analysis were investigated using 7000 Shimadzu 2 kW model X-ray spectrophotometer (Shi-madzu, Tokyo, Japan) with nickel-filtered Cu radiation (CuKα) with λ = 1.54056 Å.

### 2.3. Extraction Methods

**Scheme 1:**
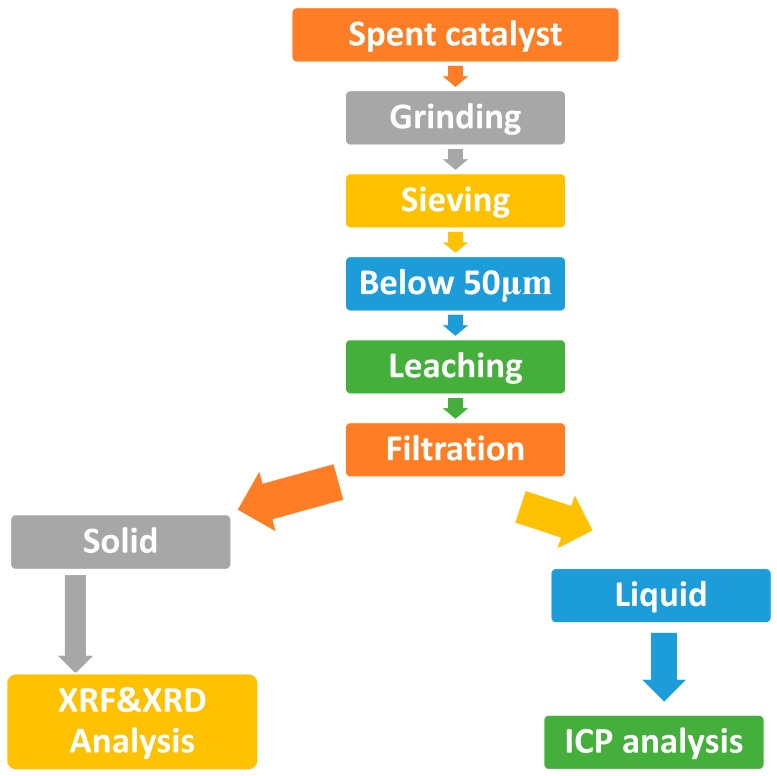
Schematic representation of the steps of sample treatment and following analysis.

Extraction strategies involved using an acidic/or basic medium for the extraction of V_2_O_5_ from the spent vanadium acid. Citric acid, oxalic acid, sulfuric acid, acetic acid, and potassium hydroxide were used for the extraction of V_2_O_5_. Each leaching experiment was performed (triplet) at the same time under the same conditions by the same person using 250 mL and/or 500 mL clean and dry Erlenmeyer flasks. These flasks were filled with different measured masses of spent catalysts varying from 5.00 to 25.00 g and a specific volume of the leaching acid or base measured by using the graduated cylinder that was added to them; then, these flasks were stirred using multi-stage hot plate with a magnetic stirrer (Witeg, Wertheim, Germany). Then, these flasks were filtrated using vacuum filtration to collect both the solid and the resulting leaching solution. The effect of experimental conditions, such as the concentration of the leaching (2–10%), exposure time (1H–4H), stirring rate (300–900 rpm), temperature (25–75 °C), and solid-to-liquid (S/L) (g of spent catalyst/mL of leaching acid or base) ratio (1/10–4/10), on the percent recovered were investigated. A schematic representation of the steps of sample treatment and the following analysis are shown in Scheme 1.

Concerning the column leaching, the column used in the leaching of the spent catalyst was made of glass (150.0 cm height and 5.0 cm internal diameter) fitted with glass wool plugs at the base to retain materials in the column. About 2.40 kg of the dried, spent catalyst was filled in the column. A total of 4.50 L of 6% oxalic acid solution was poured into the column and allowed to equilibrate for 24 h. Liquid leached samples were drained from the column every 30 min and analyzed for their content of vanadium and some other metals by using ICP. Fresh 6% oxalic acid was added to the column to keep it filled and all the spent catalysts were covered with solution. No fresh water was added.

After eluting the last solution sample from the column, a solid sample from the column was taken and analyzed via XRD and XRF.

The leaching efficiency was evaluated via the determination of the concentration of vanadium in the solution and relating the recovered vanadium to the nominal value contained in the solid using Equation (1).

(1) $$\%Recovery\;of\;Vanadium=\frac{Vanadium\;Concentration\;in\;the\;solution\times V}{mass\;of\;the\;V-ion\;in\;the\;original\;sample}\times 100\%$$

where V is the volume of the leaching solution (in mL). The concentration of vanadium in the solution was determined via ICP (in ppm).

In batch experiments, ground spent vanadium catalyst was used, but the unground spent vanadium catalyst was used in column experiments.

To minimize errors in the data and to optimize the experimental results, three equivalent runs (*n* = 3) (triplet run) and a data error analysis and statistical analysis with ANOVA test were performed.

## 3. Results and Discussion

### 3.1. Characterization of the Spent Vanadium Catalyst

**Figure 1:**
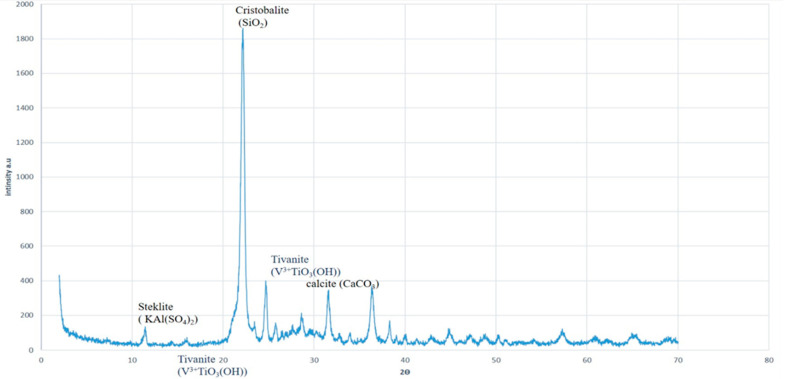
The X-ray powder diffractogram of active vanadium catalyst supplied by the Jordanian Phosphate Mines Company (Maan, Jordan). Experimental conditions: particle size ≤ 50 µm, λ = 1.54056 Å.

**Figure 2:**
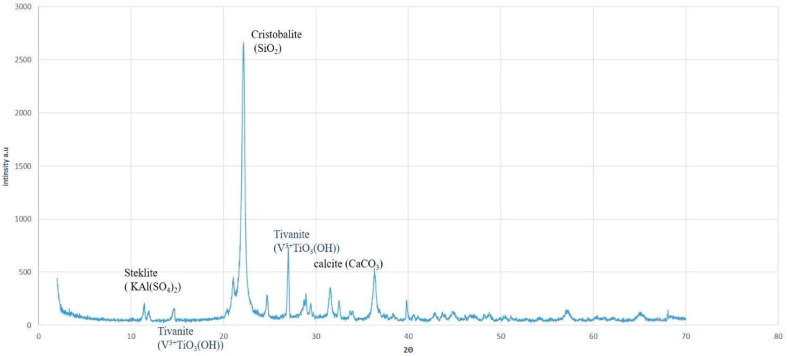
The XRD powder diffractogram of spent vanadium catalyst supplied by the Phosphate Mines Company. Experimental conditions: particle size ≤ 50 µm λ = 1.54056 Å.

The spent catalyst zeta potential (ζ) was −6.05 ± 0.05 (mV); these low zeta potential values suggest that low coagulation was achieved in the solution, while the XRD data are mentioned in Figure 1 and Figure 2. Figure 1 shows the XRD diffractogram for the active vanadium catalyst (before the industrial process) while Figure 2 shows the diffractogram for the spent vanadium catalyst (after the industrial process).

The two diffractograms displayed in Figure 1 and Figure 2 for the active and the spent catalyst show that a marginal crystallographic change occurred in the catalyst upon use in the sulfuric acid production industry. The major peaks found at 2Θ 10–12° for steklite (KAl(SO_4_), 15–17° and 22–25° for tivanite (V^3+^TIO_3_(OH) reveal the presence of vanadium, 20–23° for cristobalite (SiO_2_), and 28–30° for calcite (CaCO_3_). The absence of any noticeable change in Bragg peaks indicates that there are no major differences between the spent vanadium catalyst and the active catalyst in terms of the crystallographic structure. This result is comparable with the Zhang study that shows the Bragg diffraction of the V_2_O_5_/TiO_2_ catalyst [27]. Apparently, as expected, the catalyst loses its activity because of poisoning without any major change in its chemical structure. This conclusion is reinforced with the results of XRF, displayed in Table 1, which shows that there is a marginal difference between the active and spent vanadium catalysts.

The XRF data were used as a reference for the succeeding extraction experiments, while the calculation of V_2_O_5_ recovery depended on Equation (1) and an optimization of the experimental conditions for maximum recovery.

### 3.2. Effect of Experimental Conditions on Extraction Recovery

#### 3.2.1. The Effect of Acid Type and Concentration on Extraction Recovery

A batch acid extraction experiment was conducted using two concentrations of four different acids (50.00 mL of the acid, 5.00 g of spent catalysts, 300 rpm, 50.0 °C). These acids were citric, oxalic, sulfuric, and acetic acids, while the chosen concentrations of each acid were 2 and 4% (*w*/*w*). The acids and the selected concentrations were chosen based on economic and environmental conditions and the expected extraction efficiency.

**Figure 3:**
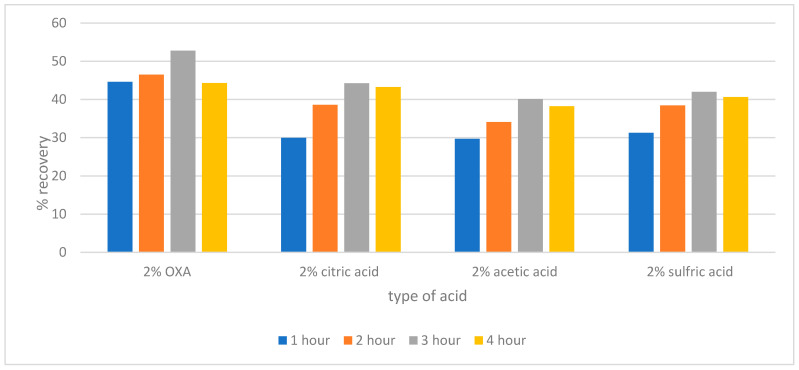
% Recovery of V-ion recovered versus the type of acid (2% *w*/*w*) after leaching.

**Figure 4:**
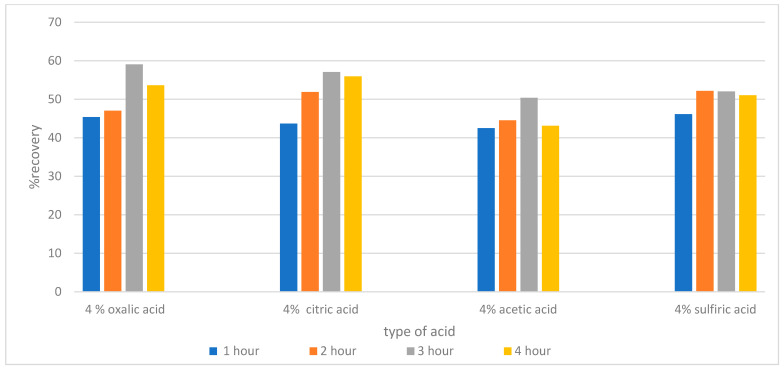
% Recovery of V-ion versus the type of acid (4% *w*/*w*) after leaching.

**Figure 5:**
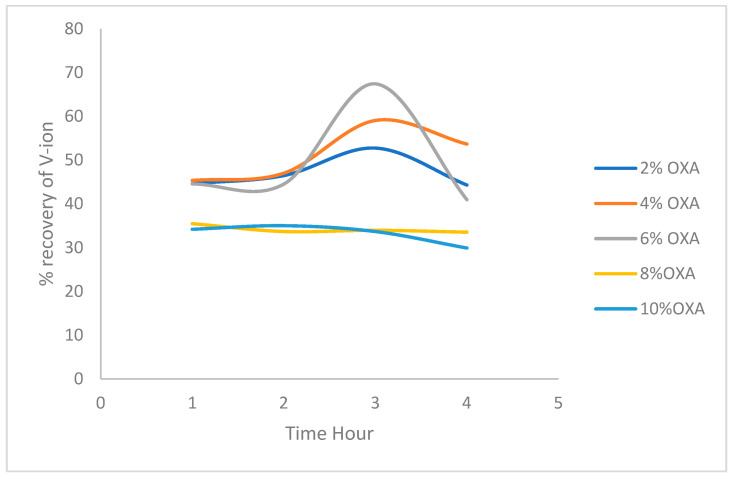
% Recovery of V-ions recovered at different times for different oxalic acid *w*/*w*% concentrations. (Color captions are shown on the right of the figure.).

The results of the effect of the acid type and concentration are displayed in Figure 3 and Figure 4. The figures show the peak time for all investigated acids and the two tested concentrations. The results also indicate that oxalic acid was the best leachant among the tested solutions since it led to a higher % recovery compared to the other acids. It is also a cheap, available, and less vigorous acid. This may be a reason for the strong interaction between V-ion and oxalate ion. The statistical treatment using the ANOVA test (an ANOVA test is a statistical test used to determine if there is a statistically significant difference between two or more categorical groups by testing for differences between means using a variance) of the data showed that there is a significant difference between the results, and the null hypothesis is rejected at *p* = 0.5 if *p*-values are used. It is worth mentioning that the null hypothesis is a significance test that is used to test the trueness of the variation in experimental data. The null hypothesis assumes with a certain probability that the variation between experimental data is due only to random errors. A positive significance test indicates with a certain probability (*p*) that the null theory is correct, and the observed variation in data is due to random errors, while a negative test indicates that the null theory is not valid and the observed variation in the data is due to real differences in the measured quantities (The *p*-value is a number calculated from a statistical test that describes how likely you are to have found a particular set of observations if the null hypothesis were true). This analysis means that oxalic acid gives the best recovery (%) compared to other acids, and oxalic acid is better than the other acids that were used. The decay in leaching efficiency after 3 h might be attributed to another competing mechanism, like the readsorption of the vanadium species on the spent catalyst frame according to the diffusion and concentration differences between the solid and liquid [23]. Figure 5 shows that leaching with 4% oxalic acid is superior to leaching with 2% oxalic acid, and this can easily be explained on a thermodynamic basis where, at a higher concentration of reactants, the equilibrium position will be shifted to the right (i.e., towards a higher degree of completion of the reaction).

#### 3.2.2. The Effect of Oxalic Acid Concentration on Leaching Efficiency

An additional investigation into the effect of changing the leachant acid concentration on the leaching efficiency was undertaken by evaluating the leaching efficiency across different concentrations of oxalic acid. A succession of oxalic acid concentrations—specifically, 2%, 4%, 6%, 8%, and 10% (*w*/*w*)—was meticulously formulated and subsequently employed for the purpose of vanadium leaching from the expended vanadium catalyst using 5.00 g of spent catalysts with a stirring rate of 300 rpm and temperature of 50.0 °C. Figure 5 effectively portrays the extent of leached vanadium in relation to the oxalic acid concentration.

**Figure 6:**
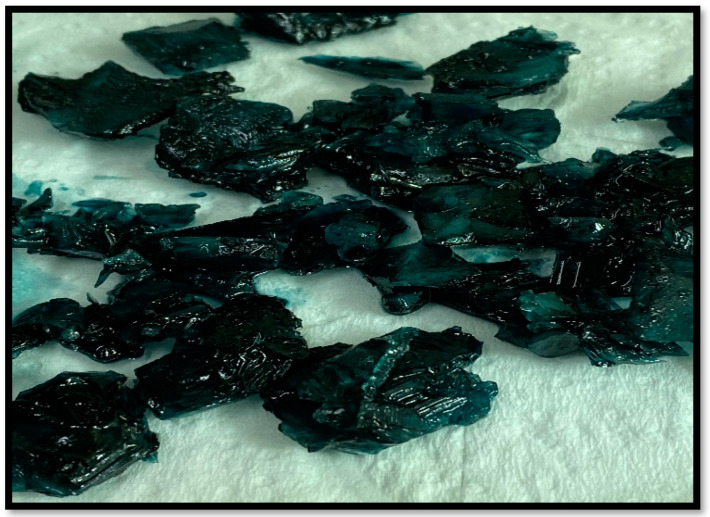
The oxalate complex that precipitated during our work.

The results displayed in Figure 5 show mostly a peak at 3 h of leaching time, as indicated previously. The highest peak value is for the concentration of the acid of 6% (*w*/*w*) oxalic acid concentration. The decline in the concentration of leached vanadium might be attributed to the formation of insoluble oxalic acid–vanadium complexes. This explanation is supported by the observation of a black precipitate (which will be characterized in a future work) in the solution shown in Figure 6 upon using higher concentrations (8% and 10%) of oxalic acid [28]. It is worth mentioning that the ANOVA statistical analysis of these results indicated a significant difference between the results, and that the null hypothesis is rejected at *p* = 0.05 so that 6% of oxalic acid concentration is used as the optimum concentration compared to the other concentrations.

#### 3.2.3. The Effect of Stirring Rate

**Figure 7:**
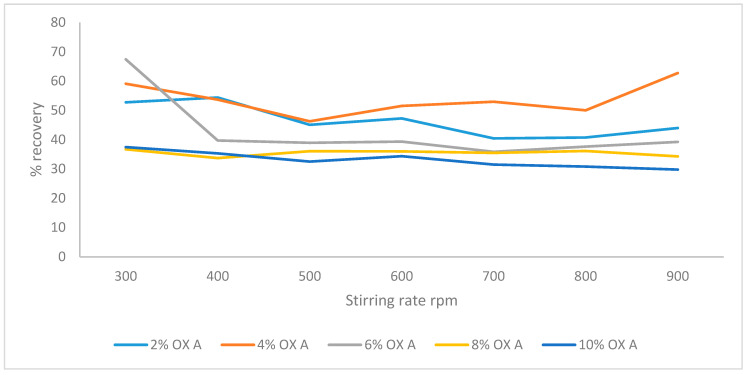
The percent recovery of vanadium versus different stirring rates with different concentrations of oxalic acid.

Figure 7 shows the effect of the stirring rate on the vanadium extraction efficiency at a constant mass of spent vanadium catalyst (5.00 g), extraction time (3 h), and volume of oxalic acid solution (50 mL). The results indicate that the optimal stirring rate is 300 rpm for almost all the investigated concentrations.

These results are attributed to the fact that a lower stirring rate allows for better contact between the solvent and the materials inside the pores in the spent porous catalyst. A higher rate of stirring causes the readsorption of the V-ion into the solid from the liquid [29]. In the literature, especially the studies of Mazurek in Poland, there is no mention of variations in stirring rate conditions; they just used 300 rpm as the optimum without listing the values of different rates [29].

#### 3.2.4. The Effect of Solid-to-Leachant Volume (S/L Ratio)

**Figure 8:**
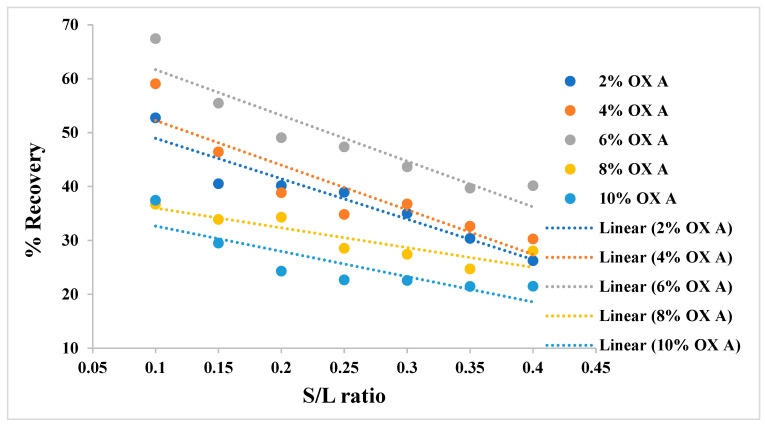
The percent recovery of vanadium ion versus different S/L ratios of spent catalysts with different oxalic acid concentrations.

Figure 8 shows the effect of the S/L ratio on the percent recovery of vanadium using five different oxalic acid concentrations (300 rpm, 50.0 °C).

The results indicate that the highest recovery was obtained at the smallest S/L ratio. These results are in general agreement with the leaching efficiency of similar systems [30,31]. In general, the leaching rate depends on several factors, such as the temperature, the size of the solid particles, the solvent used for leaching, and the diffusion (movement) of the leached ions towards the bulk of the solution. Increasing the S/L ratio will, in general, decrease the diffusion due to the increased viscosity. This will result in a hindrance in the movement, leading to a decreased leaching efficiency. This also could be explained using Equation (1), shown above: There is an increase in the mass of V_2_O_5_ in the spent catalysts as the mass of the spent catalyst increases; however, on the other hand, the mass of V_2_O_5_ in the spent catalyst is in the denominator of Equation (1). It is clear that the ppm of V recovered from the tested sample is multiplied by the volume of the recovered sample after leaching (Vf) occurs in the nominator, so the percent recovery decreases as a result of the increases in the S/L ratio since the denominator increases as the mass of the spent catalyst increases [30,31]. 

#### 3.2.5. The Effect of Temperature on Vanadium Recovery from the Spent Catalyst

**Figure 9:**
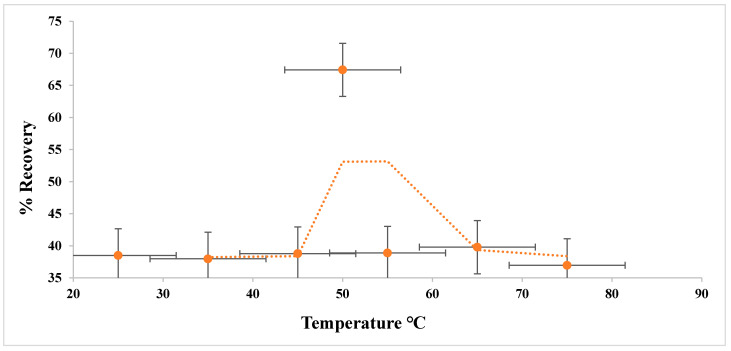
The percent recovery of vanadium ion versus temperature.

The effect of temperature on the recovery of vanadium from a spent vanadium catalyst was investigated via the determination of the percent recovery as a function of the extraction temperature between 25 °C and 75 °C under identical experimental conditions. Figure 9 shows that the temperature in the investigated range has little influence on the percent recovery except at 50 °C. ANOVA statistical treatment of the data shows that the null hypothesis is rejected at *p* = 0.05, and there is a significant difference in recovery at 50 °C, while there is an insignificant effect on recovery at other temperatures, and the null hypothesis is valid at all investigated temperatures except 50 °C at *p* = 0.05. This temperature is the same as the temperature chosen in the studies in Poland and Turkey [19,29] where it was mentioned that the effect of temperature on the leaching yield of the vanadium compounds is much smaller when leaching in an acidic environment than in an alkaline environment, while 50 °C was the optimum temperature for both cases without further explanation. From our perspective, this increase in recovery at 50.0 °C may be attributed to the coagulation of the solid spent catalyst in addition to the effect of mass transfer with diffusion factors. This might be explained by two contradicting factors: One is the increased dynamics of the system, which tends to increase the percent recovery with increasing temperature, and the other is the tendency of coagulation and agglomeration of the solution contents, which hinders the leaching of vanadium from the spent catalyst [29,32].

### 3.3. Column Leaching Experiments

**Figure 10:**
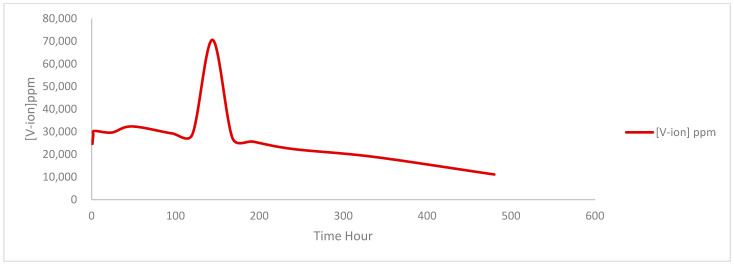
Vanadium ion concentration recovered from column versus different times using 6% oxalic acid solution.

Column-leaching experiments were performed on the spent vanadium catalyst (not grinded) packed in the column, as described in the experimental section. The composition and structure of the used spent catalyst were identical to those used in the batch experiments, as evidenced by the XRD and XRF techniques. Figure 10 shows the results of the column extraction of vanadium from the spent catalyst with 6% oxalic acid solution as a function of time. Liquid samples of 50.00 mL were collected at 30 min. intervals after 24 h equilibration time from the time that the eluted solution was introduced to the column. Extraction efficiency shows a peak after 144 h (about 6 days) (Figure 10).

**Figure 11:**
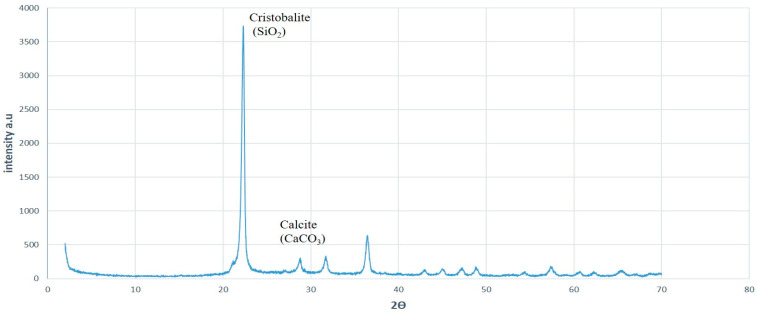
The XRD diffraction pattern for CSC.

Extraction efficiency was examined via XRD and XRF analysis of the spent vanadium catalyst after 6 days of column extraction. The results are displayed in Figure 11 and Table 2. The XRD diffractogram (Figure 11) shows the absence of the tivanite (V^3+^TiO_3_(OH)) peak, which indicates almost total extraction of vanadium from the spent catalyst. XRF (Table 2) shows that the residual V_2_O_5_ in the spent vanadium catalyst is only 0.30% compared to an initial value of 5.31%. This result attests to the efficiency of the column extraction.

### 3.4. Alkaline Leaching Experiments

**Figure 12:**
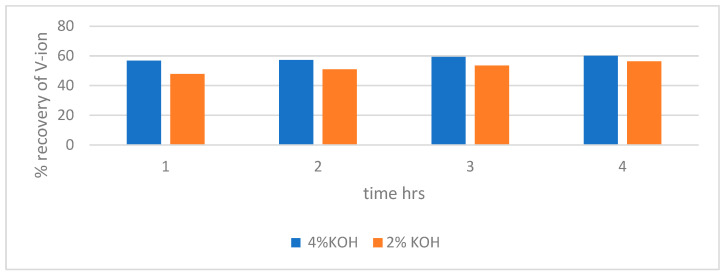
The percent recovery of vanadium ion versus time (leaching using KOH).

The alkaline leaching of vanadium ions from the vanadium-spent catalyst was also investigated. KOH was the only base used to leach vanadium ions from the spent catalyst, noting that alkaline leaching is not preferred because most metals tend to precipitate in basic solutions. In addition, leaching from basic solutions is expensive and economically unfavorable, as shown in Figure 12, which uses 50 mL of 2% KOH and 4%KOH *w*/*w*%, 5.0000 g of spent catalysts, 300 rpm, 50.0 °C. The alkaline leaching leads to a higher [V] ion recovery compared with some investigated acids; however, in this research, oxalic acid is still superior to KOH as a leacher [17,18].

## 4. Conclusions

In the present work, a methodology for the extraction of vanadium from a spent vanadium catalyst used in a sulfuric acid production plant in Jordan was proposed and optimized. Our results provide simple and safe remediation with an almost environmentally friendly procedure for the recovery of V_2_O_5_ from spent catalysts used in the Jordanian industry. In this approach, oxalic acid was used with 6% (*w*/*w*) and a 300 rpm stirring rate at 50.0 °C for 3.0 h with 2525 ppm of V-ion recovered in the solution and a percent recovery of 80.58% based on the XRF results. Also, this leaching solution was used for both batch experiments and column experiments. The latter took place over 6 days (144 h), leading to a concentration of recovered vanadium ion equal to 70,620 ppm with a percent recovery of 67.43%. This remediation can be considered a successful approach to the removal of hazardous materials from the environment. Reusing these materials in a beneficial project can open the door to the brand-new application of spent catalyst remediation. The optimization involved the selection of the leaching agent. The leaching agent that showed the highest recovery was a 6% oxalic acid solution. The optimized experimental conditions using 6% oxalic acid solution in batch experiments were 50 °C, a 300 rpm stirring rate, a 0.1 S/L ratio, and 3 hours of extraction time. The highest recovery achieved under the optimized conditions was 67.43%.

Column experiments, on the other hand, lasted for six days, reaching the maximum recovery (94.42%) using 6% oxalic acid under ambient conditions and a gravity-driven flow rate, which is a very encouraging condition for routine leaching and large-scale leaching. Obviously, the extraction efficiency achieved via column extraction requires a longer extraction time and different experimental conditions.

These results lay the foundation for further studies on the extraction of vanadium from hundreds of tons of accumulated spent catalysts and could help to save the environment using an economically acceptable methodology.

## Figures and Tables

**Table 1:** Percent (*w*/*w*) composition of active and spent vanadium catalyst as determined via X-ray fluorescence spectrometry (XRF). The data were obtained using lab center XRF-1800 (Shimadzu, Tokyo, Japan) (N.D = not detected).

Constituents	Composition (*w*/*w*) %		Constituents	Composition (*w*/*w*) %	
	Active Vanadium Catalyst	Spent Vanadium Catalyst		Active Vanadium Catalyst	Spent Vanadium Catalyst
SiO_2_	64.3229	67.8672	P_2_O_5_	0.2751	0.2184
SO_3_	13.3427	11.4236	MgO	0.2198	0.1091
K_2_O	12.1145	11.0965	MnO	0.0132	0.0080
V_2_O_5_	5.5119	5.3085	NiO	0.0081	0.0080
Al_2_O_3_	1.5307	1.5182	CuO	0.0067	0.0049
Fe_2_O_3_	1.1006	0.9865	ZnO	0.0062	0.0055
Na_2_O	0.8150	1.0137	SrO	0.0031	0.0040
CaO	0.7265	0.4279	Rb_2_O	0.0030	N.D

**Table 2:** XRF data for CSC (N.D = not detected).

Constituents	*w*/*w*% in CSC	Constituents	*w*/*w*% in CSC
SiO_2_	97.5526%	Na_2_O	0.0669%
SO_3_	0.7799%	CaO	0.0302%
K_2_O	0.7623%	P_2_O_5_	0.0479%
V_2_O_5_	0.2964%	NiO	0.0106%
Al_2_O_3_	0.3269%	CuO	0.0028%
Fe_2_O_3_	0.1104%	MgO, MnO	N.D

## Data Availability

All data analyzed during this study are included in this published article.
